# Medical students’ patterns of using ChatGPT as a feedback tool and perceptions of ChatGPT in a Leadership and Communication course in Korea: a cross-sectional study

**DOI:** 10.3352/jeehp.2023.20.29

**Published:** 2023-11-10

**Authors:** Janghee Park

**Affiliations:** Department of Medical Education, Soonchunhyang University College of Medicine, Cheonan, Korea; Hallym University, Korea

**Keywords:** Artificial intelligence, Feedback, Leadership, Medical students, Republic of Korea

## Abstract

**Purpose:**

This study aimed to analyze patterns of using ChatGPT before and after group activities and to explore medical students’ perceptions of ChatGPT as a feedback tool in the classroom.

**Methods:**

The study included 99 2nd-year pre-medical students who participated in a “Leadership and Communication” course from March to June 2023. Students engaged in both individual and group activities related to negotiation strategies. ChatGPT was used to provide feedback on their solutions. A survey was administered to assess students’ perceptions of ChatGPT’s feedback, its use in the classroom, and the strengths and challenges of ChatGPT from May 17 to 19, 2023.

**Results:**

The students responded by indicating that ChatGPT’s feedback was helpful, and revised and resubmitted their group answers in various ways after receiving feedback. The majority of respondents expressed agreement with the use of ChatGPT during class. The most common response concerning the appropriate context of using ChatGPT’s feedback was “after the first round of discussion, for revisions.” There was a significant difference in satisfaction with ChatGPT’s feedback, including correctness, usefulness, and ethics, depending on whether or not ChatGPT was used during class, but there was no significant difference according to gender or whether students had previous experience with ChatGPT. The strongest advantages were “providing answers to questions” and “summarizing information,” and the worst disadvantage was “producing information without supporting evidence.”

**Conclusion:**

The students were aware of the advantages and disadvantages of ChatGPT, and they had a positive attitude toward using ChatGPT in the classroom.

## Graphical abstract

[Fig f2-jeehp-20-29]

## Introduction

### Background/rationale

The term artificial intelligence (AI) encompasses not just the technology itself, but also the cognitive processes through which computers process information similarly to humans [1]. ChatGPT (GPT: generative pre-trained transformer) is a large language model launched on November 30, 2022 [2]. ChatGPT stands out from traditional internet services with its ability to provide interactive and personalized information for individuals based on pre-trained data.

Apart from offering optimized answers to questions, it also summarizes searched articles or existing materials, generates text, and provides diverse feedback. As a result, many people have quickly adopted and utilized this AI chatbot. One of its key advantages is its ability to converse in various languages and provide quick translations into different languages. Due to these capabilities, it is widely used in academic work, including information retrieval and summarization, and it has even raised debates about AI-authored papers and copyright issues. In education, it aids in diverse information searches, offers personalized feedback on learning outcomes, contributes to learning enhancement, and encourages self-directed learning [2-4].

In education, feedback is utilized as an essential strategy to promote individualized and continuous growth, alongside teaching and assessment. For feedback to be effective, it should be routine, timely, non-threatening, specific, and encourage self-assessment [4]. However, in cases where there are a large number of students, it can be challenging for instructors to provide prompt feedback on assessments or assignments. Automated scoring programs systematically evaluate input using well-defined rubrics and offer feedback based on these evaluations. This feedback is programmed to achieve intended outcomes, enhancing students’ learning capabilities [5]. However, achieving this necessitates additional efforts and costs, such as the resources required for program utilization. Considering its accessibility to the general public, ChatGPT was explored as a means of providing feedback in classes.

### Objectives

The purpose of this study was to analyze patterns of using ChatGPT before and after group activities and explore medical students’ perceptions of ChatGPT as a feedback tool in the classroom.

## Methods

### Ethics statement

The survey included a question related to informed consent.

### Study design

This was a cross-sectional study based on a questionnaire survey after the use of ChatGPT as a feedback tool.

### Setting

**Fig. 1.**
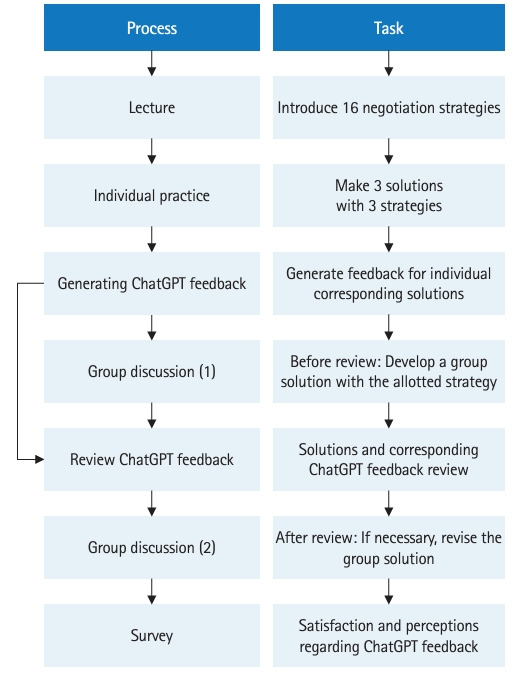
The research process.

The research process is shown in the flowchart in Fig. 1. In the first semester (March to June) of 2023, a course titled “Leadership and Communication” was conducted for 2nd-year pre-medical students. During the course, 16 negotiation strategies were introduced in the lecture. Students practiced these negotiation strategies as an individual activity, in which they made 3 solutions with 3 strategies among 16 negotiation strategies to solve a simulated conflict scenario. At first, students submitted 3 solutions in a physical format on paper, but they subsequently resubmitted them along with a text file to generate feedback from ChatGPT. In the first session of team learning, each team developed a team solution with the allotted negotiation strategy without feedback from ChatGPT. After completing the first session of team learning and submitting the solution, the instructor presented the feedback from ChatGPT for the corresponding solution. Each group reviewed and discussed the feedback, and revised and resubmitted their solutions if necessary. After finishing the group activity, a survey was conducted from May 17 to 19, 2023 regarding satisfaction with ChatGPT’s feedback and perceptions of ChatGPT, including its strong and weak points. The anonymous survey was conducted using the e-class system, which is a program developed at Soonchunhyang University similar to a learning management system. The 16 negotiation strategies, the simulated conflict scenario, and the tasks of activities are available in Supplement 1.

### Participants

The participants were 99 2nd-year pre-medical students engaged in developing solutions to resolve simulated conflict scenarios during both individual and group activities at Soonchunhyang University, Korea.

### Variables

The outcome variables were participants’ opinions about ChatGPT’s feedback.

### Data sources/measurement

The feedback from ChatGPT for students had limitations regarding the word count of questions, so we made a standardized question format and asked repeatedly. The overall structure specified the feedback condition, problem, scenario, and the student’s solution list.

Students’ opinions on ChatGPT’s feedback were assessed using a Likert scale, where participants were asked to rate their responses on the following scale: strongly agree (5), agree (4), neutral (3), disagree (2), and strongly disagree (1). The strengths and weaknesses of feedback from ChatGPT were evaluated through a multiple-response format, and the appropriate timing for utilizing the feedback in a group discussion class was determined. The survey questionnaire about the feedback from ChatGPT is available in Supplement 2. The reliability of the satisfaction items (correctness, helpfulness, ethicality) was high (Cronbach α=0.817). The correlation between correctness and helpfulness was also high (r=0.914).

### Bias

Out of 99 target students, only 42 students responded voluntarily. There may have been bias due to an excessive number of non-responding students.

### Study size

No prior sample size was estimated for analyzing the outputs by individual and group activity in the same class, because all target students were invited to participate.

### Statistical methods

Frequency analysis and the Mann-Whitney U test were performed. Statistically significant differences were expressed as P<0.05. All data were processed in IBM SPSS version 27.0 (IBM Corp.).

## Results

### Participants

Ninety-nine students in a class participated in both the individual and group activities. After class, 42 students responded to the anonymous online survey, but one respondent did not agree with the informed consent, so data from 41 respondents (23 men, 18 women) were analyzed.

### Patterns of using ChatGPT before and after group activities

Students utilized ChatGPT’s feedback effectively (Table 1). In individual activities, students selected 3 out of 16 negotiation strategies and developed 292 solutions based on their chosen strategies (Dataset 1). The mean number of solutions per strategy was 18.25. The most commonly selected strategy was Strategy 8 (ask many questions when negotiating), chosen by 49 students, while the least selected strategy was Strategy 11 (remember that having more negotiation authority doesn’t necessarily make your negotiation power greater), chosen by only 4 students. In the group activity, all 15 teams developed solutions for minimizing the conflict with the allotted negotiation strategy. The 16th strategy (if the other party is in a disadvantageous position in negotiations, express regret instead of apologizing) was not discussed in the group activity for efficient management.

Out of 15 teams, 13 utilized ChatGPT’s feedback and resubmitted revised solutions by adding conclusions (5 teams), extra explanations (4 teams), or producing new strategies (3 teams). In the 9th team, students voluntarily received additional feedback from ChatGPT and submitted a new solution. Two teams did not revise their solutions.

### Use of ChatGPT as a feedback tool

After the group activity, a survey was performed about satisfaction and perceptions of feedback from ChatGPT (Dataset 2 and Tables 2, 3). Students responded regarding the correctness and helpfulness of ChatGPT’s feedback with an average score of approximately 3.5 out of 5. The perceived “ethicality of feedback content” received a relatively high rating of 4.05. Seventy-six percent of the students had previously utilized and/or encountered information related to ChatGPT. The majority of respondents expressed agreement with the utilization of ChatGPT during class.

Regarding the appropriate timing for employing ChatGPT’s feedback during discussion sessions, the responses were as follows: “after the first round of discussion, for revisions” (42.9%), “during the discussion, as a reference” (33.3%), and “after the discussion ends, for reference purposes” (23.8%). No one selected “before the discussion, as a preview.”

The degree of satisfaction with feedback from ChatGPT (Table 4), including correctness, helpfulness, and ethicality, showed significant difference according to whether ChatGPT had been used in class, but no significant differences according to either gender or previous ChatGPT user.

### The advantages and disadvantages of ChatGPT

The 31 students who had used ChatGPT before or encountered related information (question 4) responded on the advantages and disadvantages of ChatGPT (Tables 2, 3). In terms of the advantages of ChatGPT, the results of multiple responses indicated that “providing answers to questions” (58.1%) and “summarizing information” (51.6%) were the most commonly chosen options. “Receiving feedback on work” and “using images” received fewer responses.

Regarding the disadvantages of ChatGPT, responses to the multiple-response questions indicated that “produces information without supporting evidence” (54.8%) and “offers general responses, lacking detailed descriptions” (19.4%) were the main concerns.

## Discussion

### Key results

The students responded by indicating that ChatGPT’s feedback was helpful, and they revised and resubmitted their group answers in various ways after receiving feedback. The majority of respondents expressed agreement with the utilization of ChatGPT during class. The most common response concerning the appropriateness of using ChatGPT’s feedback was “after the first round of discussion, for revisions.” In terms of advantages, “providing answers to questions” and “summarizing information” were the most frequently mentioned. As for disadvantages, the most prevalent view was that ChatGPT “produces information without supporting evidence.” Students who responded that ChatGPT’s feedback had high accuracy and helpfulness exhibited a positive response toward using ChatGPT during class time.

### Interpretations

Students were already aware of the advantages and disadvantages of ChatGPT, and considering these pros and cons, they had a positive attitude toward utilizing ChatGPT in the classroom. Most students had been using or were well aware of ChatGPT for the past 5 months; ChatGPT was launched in December 2022, and the survey was conducted in May 2023. The primary advantage of ChatGPT, as perceived by the students, was its ability to provide answers to questions and summarize information. On the other hand, the most significant disadvantage was the potential provision of inaccurate information or relying on past knowledge.

In general, students expressed a consensus in favor of incorporating ChatGPT into the classroom. The more students perceived ChatGPT’s feedback as accurate and helpful, the more they expressed agreement with its utilization during class. However, there were also negative opinions regarding the feedback being too conventional, suggesting a need for improvement. Whether students had prior experience with ChatGPT or preexisting knowledge did not show statistically significant relationships with their classroom usage, indicating that their opinions on the feedback, rather than bias, influenced their decision to use ChatGPT during class. There were differing views regarding the optimal timing for its integration during class sessions. In the context of discussion-based classes, the preferred approach was to employ ChatGPT for reference and feedback after the conclusion of the discussion.

### Comparison with previous studies

There are 3 primary categories of articles related to ChatGPT. The first category comprises introductions to GPT, which typically cover its historical background, advantages and disadvantages, and usage guidelines. [1,4-7]. The results of this study align with the conclusions presented in other articles addressing the strengths and challenges associated with ChatGPT. ChatGPT provides specific responses to queries, but it is essential to scrutinize the accuracy of these answers by verifying the supporting evidence.

The second category involves the assessment of ChatGPT’s knowledge accuracy through testing, including examinations such as United States Medical Licensing Examination, the Situational Judgement Test, and subject tests in medical school [8-12]. In this study, the majority of the students who responded regarding the feedback provided by ChatGPT stated that it demonstrated a high degree of accuracy.

The third category is centered around the examination of ChatGPT’s utilization within educational settings, including the classroom. An attempt was made to develop ChatGPT in order to design suitable scenarios, facilitate simulated patient-physician role-play, and provide real-time feedback to the physician user [13]. This study explored the application of ChatGPT as a feedback tool. It is worth noting that there is limited existing research directly implementing ChatGPT in medical education involving students as the subjects. The primary distinctions from previous articles were the utilization of ChatGPT as a feedback tool in the classroom to alleviate the educator’s workload and the administration of a survey assessing students’ perceptions of ChatGPT’s feedback.

### Limitations

This study utilized ChatGPT version 3.5 to provide feedback on students’ responses in May 2023. It is worth noting that ChatGPT is continuously being upgraded, and the availability of various large language models may lead to differing feedback outcomes when using different versions or models at different times.

### Suggestions for further studies

AI-related programs will continue to advance in the future and can be utilized in diverse ways in medical education. As educators and end-users, it is imperative to actively engage with AI-related programs from their initial stages, offering insights into future development directions [2,6]. To address the constraints associated with ChatGPT and to employ it effectively within medical education, collaboration among educators, researchers, and practitioners is essential. They must collectively formulate best practices, guidelines, and policies. This endeavor requires sustained research efforts and interdisciplinary cooperation [7,14].

In this study, students’ handwritten responses were transcribed into a digital format to obtain feedback from ChatGPT. It is advantageous to receive ChatGPT’s feedback by providing students’ assignments as a text file. Furthermore, rather than instructors individually providing feedback results to each student, a more effective approach may involve students submitting their responses along with ChatGPT’s feedback results. In this way, instructors can provide comprehensive feedback on similar feedback patterns, potentially increasing student engagement while reducing the instructor’s workload.

### Conclusion

The most commonly selected strategy was “ask many questions when negotiating;” while, the least selected strategy was “remember that having more negotiation authority doesn’t necessarily make your negotiation power greater.” Students were satisfied with the feedback provided by ChatGPT and made revisions to their group submissions in various ways. Despite their prior awareness of the strengths and weaknesses of ChatGPT, the majority of students agreed that ChatGPT would be appropriate to use as a feedback tool in the classroom. Greater satisfaction with the feedback correlated with a higher degree of agreement regarding the use of ChatGPT in the classroom.

## Figures and Tables

**Figure f2-jeehp-20-29:**
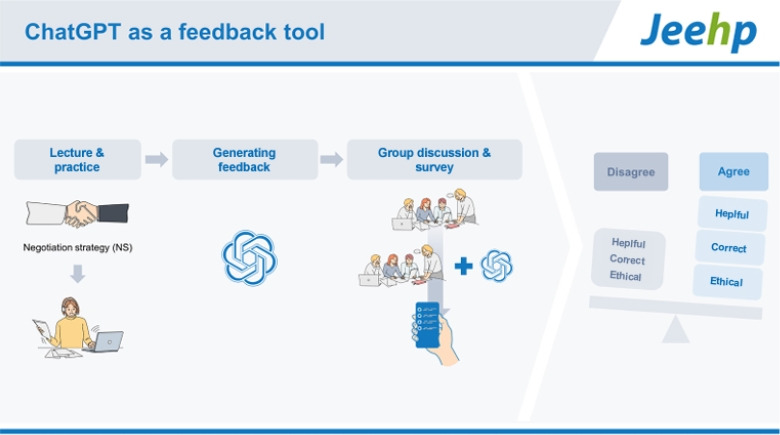


**Table 1.** Individual and group solutions for the simulated conflict scenario

	No. of solutions	Negotiation strategy no.
The individual student’s solutions		
Mean	18.25	
Standard deviation	14.1	
Max	49	8
Min	4	11
Total	292	
Groups’ solutions		
Main difference between before and after reviewing feedback		
Added concluding statements	5 (33.3)	1, 2, 8, 10, 11
Provided additional explanations	4 (26.7)	4, 5, 7, 12
Introduced a new answer	3 (20.0)	6, 14, 15
Conducted extensive revisions based on the additional feedback received from ChatGPT	1 (6.7)	9
Did not make any revisions	2 (13.3)	3, 13
Total	15 (100.0)	

Values are presented as number or number (%).

**Table 2.** The survey results regarding the utilization and perceptions of ChatGPT

ChatGPT’s feedback	Score	Students’ responses					
		Strongly disagree	Disagree	Neutral	Agree	Strongly agree	Total
Correct	3.51±1.08	2 (4.9)	6 (14.6)	8 (19.5)	19 (46.3)	6 (14.6)	41 (100.0)
Helpful	3.59±1.02	2 (4.9)	4 (9.8)	9 (22.0)	20 (48.8)	6 (14.6)	41 (100.0)
Ethical	4.05±0.64	7 (17.1)	24 (58.5)	9 (22.0)	40 (97.6)	1 (2.4)	41 (100.0)

Values are presented as mean±standard deviation or number (%).

**Table 3.** The survey results regarding the utilization and perceptions of ChatGPT

ChatGPT’s feedback	Questions	No. (%)
The usage in the class	1. Are you in favor of using ChatGPT in the classroom?	
	Yes, I support it.	31 (75.6)
	No, it should not be used in the classroom.	7 (17.1)
	No response	3 (7.3)
	Total	41
	2. When is the most effective time to provide feedback from ChatGPT during a discussion class?	
	After the first round of discussion, for revisions.	17 (41.5)
	During the discussion, as a reference.	14 (34.1)
	After the discussion ends, for reference purposes.	10 (24.4)
	Before the discussion, as a preview.	
	Total	41
Experience	3. Have you ever used ChatGPT before or encountered related information?	
	Yes	31 (75.6)
	No	10 (24.4)
	Total	41
Advantages and disadvantages of ChatGPT (only the experienced, multiple responses)	4. Which function (advantage) do you think would be most useful?	
	Providing answers to questions.	18 (58.1)
	Summarizing and organizing materials.	16 (51.6)
	Finding the latest information.	4 (12.9)
	Providing feedback on my responses.	6 (19.4)
	Drawing desired images.	1 (3.2)
	Total	45
	5. What is the most significant weakness of ChatGPT?	
	Produces information without supporting evidence.	17 (54.8)
	Offers general responses, lacking detailed descriptions.	6 (19.4)
	Provides misleading information convincingly.	4 (12.9)
	Provides answers based on pre-trained past knowledge.	3 (9.7)
	Weak in understanding emotional expressions.	3 (9.7)
	Total	33

**Table 4.** Group differences in satisfaction with ChatGPT’s feedback (Mann-Whitney U test)

Variable	Correct			Helpful			Ethical		
	No.	Median (IQR)	P-value	No.	Median (IQR)	P-value	No.	Median (IQR)	P-value
Gender			0.365			0.866			0.598
Male	23	4 (3–5)		23	4 (3–4.25)		22	4 (3.75–4.25)	
Female	18	4 (2.25–4)		18	4 (3–4)		18	4 (4–4.75)	
Q4. Pre-experience/pre-information			0.595			0.871			0.385
No	10	4 (2.50–4.75)		8	4 (2.50–4.75)		8	4 (3.25–4)	
Yes	31	4 (3–4)		31	4 (3–4)		32	4 (3–4)	
Q2.Using ChatGPT in class			0.012			0.005			0.006
No	7	2 (1–3)		7	2 (1–3)		7	3 (3–4)	
Yes	31	4 (3–4)		31	4 (3–4)		31	4 (4–5)	

IQR, interquartile range.

## Footnotes

**Data availability**
Data files are available from Harvard Dataverse: https://doi.org/10.7910/DVN/IDSD81 Dataset 1. ChatGPT’s feedback on students’ solutions (Korean language).

Dataset 2. Survey response data on the strengths and weaknesses of feedback from ChatGPT.

## Supplementary materials

Supplementary files are available from Harvard Dataverse: https://doi.org/10.7910/DVN/IDSD81

Supplement 1. The research process

Supplement 2. Survey questionnaires about ChatGPT’s feedback in Korean and translated into English

Supplement 3. Audio recording of the abstract.
