# Supplementary material for: Medical students’ patterns of using ChatGPT as a feedback tool and perceptions of ChatGPT in a Leadership and Communication course in Korea: a cross-sectional study
Source: J Educ Eval Health Prof. 2023 Nov 10;20:29. doi: 10.3352/jeehp.2023.20.29 (PMC10725745; doi:10.3352/jeehp.2023.20.29)

Supplement 1. The research process

| **Schedule** | **Leadership and Communication Course** |
| --- | --- |
| 1. Lecture | Content of lecture: The 16 negotiation strategies |
|  | 1. Don’t think that there is a fixed pie in negotiations.  2. Utilize the theory of relative value in negotiations.  3. Don’t underestimate your negotiation power.  4. Negotiation power emerges when you are prepared for adventure in negotiations.  5. Don’t propose narrowing the price difference first.  6. Recognize that the other party’s problem may not be limited to just the other party.  7. Understand what the other party wants, not just their surface position.  8. Ask many questions when negotiating.  9. Don’t mistakenly assume that knowing the concessions the other party wants in negotiations gives you an advantage.  10. In negotiations, appearing to have negotiation power often means having influence.  11. Remember that having more negotiation authority doesn’t necessarily make your negotiation power greater.  12. It’s important to preserve the other party’s dignity when necessary.  13. Don’t fixate on one negotiation method; be creative and explore different approaches.  14. People are unique, but there are predictable patterns.  15. Prepare in advance for cases where you can’t reach an agreement.  16. If the other party is in a disadvantageous position in negotiations, express regret instead of apologizing. |
| 2. Individual activity | Solve the simulated conflict scenario with 3 negotiation strategies among 16. |
|  | The simulated conflict scenario:  The deadline for submitting a group project is 2 weeks away.  I am a group leader.  It’s difficult to participate in group meetings with part-time jobs. (Teammate 1)  I am busy with club activities. (Teammate 2)  I’m not interested in getting good grades. (Teammate 3)  I need good grades to receive a scholarship. (Teammate 4) |
| 3. Generating ChatGPT feedback | Generating the ChatGPT feedbacks for each student answers (solutions) |
|  | Prompt  1. The overall structure of the text was described.  2. Explain how to feedback, such as professor level, based on the strategy.  3. The problem was expounded upon to establish the criteria for feedback.  4. Describe the conflict situated scenario.  5. List of the student’s answers (solutions) |
| 4. Group activity | Step 1 discussion: Solve the simulated conflict situation with allotted strategy among 16 strategies.  • Reference: all student’s answers |
|  | Check the ChatGPT feedback |
|  | Step 2 discussion: If necessary, discuss and change the group solution. |
| 5. Survey |  |
|  | 1. Gender  2. The feedback about the ChatGPT’s feedback (5-Likert scale)  A. Helpful?  B. Correct?  C. Ethical?  3. The usage in the class  A. Agree or disagree (yes/no)  B. Timing (Multiple Choice Questions)  4. The experience? (yes/no)  5. Perceptions about ChatGPT (only the experienced, multiple responses)  A. Strengths  B. Weakness |

**Example of prompt for generating feedback**


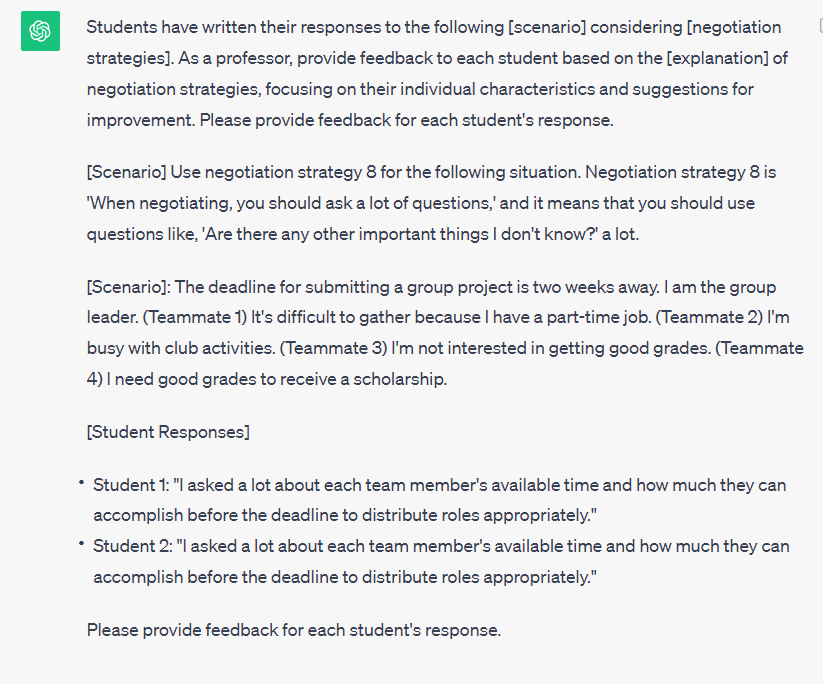

Supplement: Supplementary file 3 — Supplement 1. The research process [file jeehp-20-29-suppl1.docx]
