# Supplementary material for: Medical students’ patterns of using ChatGPT as a feedback tool and perceptions of ChatGPT in a Leadership and Communication course in Korea: a cross-sectional study
Source: J Educ Eval Health Prof. 2023 Nov 10;20:29. doi: 10.3352/jeehp.2023.20.29 (PMC10725745; doi:10.3352/jeehp.2023.20.29)
Supplement: Supplementary file 4 — Supplement 2. Survey questionnaires about ChatGPT’s feedback in Korean and translated into English [file jeehp-20-29-suppl2.docx]

Supplement 2. Survey questionnaires about ChatGPT’s feedback in Korean and translated into English

**Satisfaction with ChatCPT feedback and Perceptions of ChatGPT Utilization**

Q1. Consent inform

This survey is being conducted for research on team activities and the use of ChatGPT in education. The survey questions are broadly categorized into the respondent's characteristics, roles in team activities, and the effectiveness of ChatGPT feedback. The survey results will be coded in a way that prevents personal identification, and they will not be used for any other purposes beyond this research. After the research is concluded, the data will be destroyed. Do you consent to the use of the survey results for medical education research?

(1) Yes (2) No

Q2. Gender

(1) Male (2) Female

Q3. [Correctness] Do you believe ChatGPT accurately understands the situation and provides feedback?

(1) Strongly disagree

(2) Disagree

(3) Neutral

(4) Agree

(5) Strongly agree

Q4. [Helpfulness] Did ChatGPT’s feedback assist in understanding the correct answers?

(1) Strongly disagree

(2) Disagree

(3) Neutral

(4) Agree

(5) Strongly agree

Q5. [Ethical Considerations] Was ChatGPT’s feedback ethical?

(1) Strongly disagree

(2) Disagree

(3) Neutral

(4) Agree

(5) Strongly agree

Q6. Are you in favor of using ChatGPT in the classroom?

(1) Yes, I support it.

(2) No, it should not be used in the classroom.

Q7. When is the most effective time to provide ChatGPT feedback during a discussion class?

(1) After the first round of discussion, for revisions.

(2) During the discussion, as a reference.

(3) After the discussion ends, for reference purposes.

(4) Before the discussion, as a preview.

Q8. Have you ever used ChatGPT before or encountered related information?

(1) Yes (2) No

Q9. Which function (advantage) do you think would be most useful? (multiple response)

(1) Providing answers to questions.

(2) Summarizing and organizing materials.

(3) Finding the latest information.

(4) Providing feedback on my responses.

(5) Drawing desired images.

Q10. What is the most significant weakness of ChatGPT? (multiple response)

(1) Produces information without supporting evidence.

(2) Offers general responses, lacking detailed descriptions.

(3) Provides misleading information convincingly.

(4) Provides answers based on pre-trained past knowledge.

(5) Weak in understanding emotional expressions.

Thank you for your responses.
